# The effects of corticotomy and piezocision in orthodontic canine retraction: a randomized controlled clinical trial

**DOI:** 10.1186/s40510-021-00367-3

**Published:** 2021-10-04

**Authors:** Larissa Salgado da Matta Cid Pinto Fernandes, Daniel Santos Fonseca Figueiredo, Dauro Douglas Oliveira, Ricardo Gontijo Houara, Wellington José Rody, Bruno Frazão Gribel, Rodrigo Villamarim Soares

**Affiliations:** 1 School of Dentistry, Pontifical Catholic University of Minas Gerais, Belo Horizonte, Brazil; 2 Graduate Program in Orthodontics, School of Dentistry, Pontifical Catholic University of Minas Gerais, Avenida Dom José Gaspar, 500. Prédio 46, Sala 106, Belo Horizonte, MG 30535-901 Brazil; 3 Department of Orthodontics and Pediatric Dentistry, Stony Brook School of Dental Medicine, Stony Brook, NY USA; 4 Belo Horizonte, Brazil; 5 Periodontics Department and Dean of Graduate Studies, Pontifical Catholic University of Minas Gerais, Belo Horizonte, Brazil

**Keywords:** Corticotomy, Piezocision; Tooth movement, Gingival crevicular fluid

## Abstract

**Background:**

The aims of this study were to evaluate the efficacy of alveolar corticotomy (AC) and piezocision (PZ) in accelerating maxillary canine retraction, and their effects on multiple bone remodeling expression in gingival crevicular fluid (GCF). A split-mouth, randomized controlled clinical trial was performed at the Department of Orthodontics of Pontifical Catholic University of Minas Gerais, Brazil. Eligibility criteria included orthodontic need for first maxillary premolars extractions, followed by canine retraction. Fifty-one adult patients were recruited and randomly assigned to 3 groups (allocation ratio 1:1:1). Random allocation of surgical or control interventions to each side of the maxillary arch was also conducted: G1 − AC × Control, G2 − PZ × Control, and G3 − AC × PZ. Both the definition of the group and the decision of the experimental or control sides were randomized by the software. Intraoral digital scans were performed before, 7 and 14 days after the beginning of canine retraction, and subsequently, at every 14 days until a maximum period of 6 months. GCF samples were collected before, and 1, 2, 4, 8, and 12 weeks. The primary outcome consisted in the cumulative distal movement of the canines and was measured by digital model superimposition. The secondary outcome consisted in GCF bone remodeling samples that were quantified in a multiplex immunoassay. The measurements examinator was properly blinded.

**Results:**

Forty-seven patients, 19 males and 28 females, were analyzed (mean age 20.72, SD = 6.66, range 15 to 38). Statistically significant differences in canine distal movement between AC and control in G1 were not observed (*p* > 0.05). In G2, PZ showed lower cumulative incisal and cervical measurements than control from the 2nd to the 24th week (*p* < 0.05). In G3, PZ showed a lower cumulative incisal and cervical measurements than AC from the16th to the 24th week (*p* < 0.05). In all groups, differences on biomarkers expression occurred at specific timepoints (*p* < 0.05), but a distinct pattern was not observed.

**Conclusions:**

AC and PZ were not effective to accelerate maxillary canine retraction and did not induce a distinct pattern of biomarker expression.

**Trial registration:**

NCT03089996. Registered 24 March 2017 - Registered.

## Background

Since a common complaint among orthodontic patients is the prolonged treatment duration, alternatives aiming to accelerate orthodontic tooth movement (OTM) have been developed in the past decades [1–3]. Among these, studies investigating the use of alveolar corticotomy (AC) have gained prominence. AC consists of intentionally causing alveolar bone injuries limited to the cortical bone, which generates a cicatricial response that leads to a local transitory acceleration of bone metabolism and a decrease in bone density, a condition known as regional acceleratory phenomenon (RAP) [1]. Although this surgical procedure has been previously reported to accelerate OTM [2, 3], recent studies indicate that the level of the evidence is low [4–7].

More recently, a less-invasive technique, piezocision (PZ), got considerable attention [8]. In this surgical procedure, piezoeletric tips are used to perform short incisions in the interradicular regions of the cortical bone, without requiring mucoperiosteal flaps or sutures [8].Some studies have reported the effectiveness of PZ to accelerate OTM [9, 10]. Nevertheless, to this date, consensus regarding its effectiveness in accelerating OTM is not observed [11–13].

Therefore, the effects of AC and PZ on OTM have been a subject of ongoing debate that did not allow clinicians to make a more scientific-based decision about their true benefits. Hence, in the present randomized controlled clinical trial (RCT), a longer follow-up period, digital models superimpositions, and molecular analysis of gingival crevicular fluid (GCF) were conducted to evaluate the effects of these procedures on the underlying alveolar bone, in order to determine if these surgeries are able to accelerate maxillary canine retraction. The null hypothesis was that there would be no significant differences between canine retraction assisted by AC or PZ in comparison with the retraction carried out without any surgical procedure, as well as no difference between acceleration rate comparing AC and PZ in canine retraction.

## Materials and methods

### Trial design and any changes after trial commencement

This is a split-mouth, randomized (allocation rate 1:1:1) and controlled clinical trial, with a parallel group design. Patients who met the inclusion criteria were enrolled from the Department of Orthodontics of Pontifical Catholic University of Minas Gerais, Brazil, from February 2016 to June 2017. Changes in study methods after enrollment did not occur. This trial was registered in ClinicalTrials.gov (NCT03089996).

### Participants, eligibility criteria, and settings

**Fig. 1:**
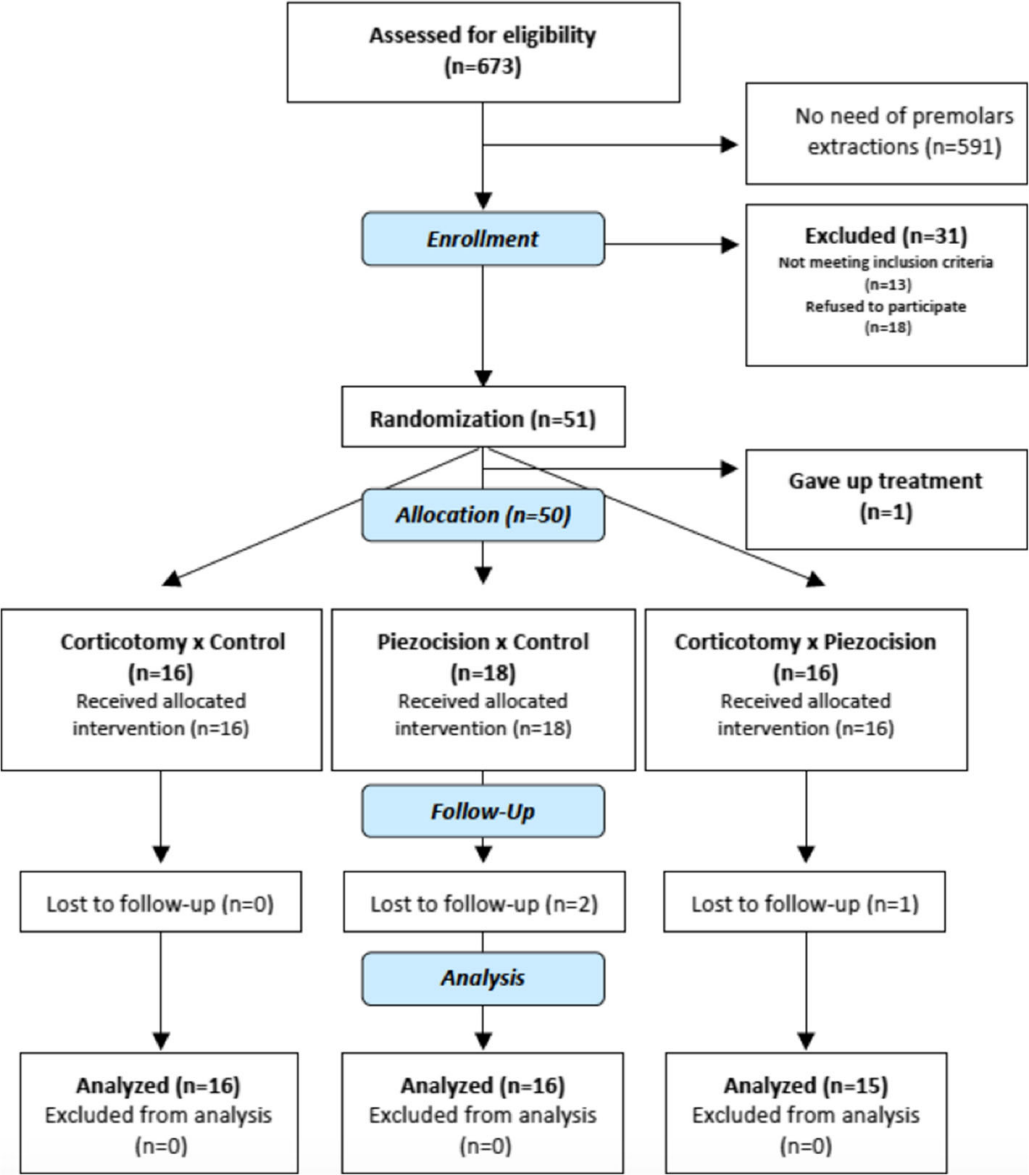
CONSORT study flowchart

The following inclusion criteria were applied: orthodontic need for first maxillary premolars extractions, age between 15 and 38 years, good oral health, presence of all upper teeth (except 3rd molars) and availability for consultation at every 2 weeks. Exclusion criteria included patients with history of periodontitis, pregnancy, patients with systemic diseases, smokers, severe crowding, presence of any craniofacial syndromes, chronic use of anti-inflammatories, and altered bone metabolism (i.e., use of antiresorptive drugs, steroids, or immunosuppressants). A total of 673 individuals were assessed for eligibility, and participants flow during the trial is described in the CONSORT flow chart (Fig. 1). Consents were obtained from all participants/parents/guardians. The study was approved by the Institutional Review Board of the Pontifical Catholic University of Minas Gerais (PUC Minas), Brazil.

### Interventions

Participants were submitted to extraction of both maxillary first premolars, 0.022 × 0.028-in orthodontic appliances were bonded (Mini-Master Series, American Orthodontics, Sheboygan, WI), and preliminary leveling and alignment was initiated. Three months after the extractions, a 0.016 × 0.022-in passive stainless-steel wire was placed, and 6-mm miniscrews (Morelli Ortodontia, Sorocaba, SP, Brazil) were inserted between the roots of the 2nd premolars and 1st molars to achieve absolute anchorage. Participants were then randomly divided into 3 groups. In group 1 (G1), AC was implemented on the experimental side, with the contralateral side being used as control. In group 2 (G2), PZ was implemented on the experimental side, with the contralateral side being used as control. Group 3 (G3) comprised individuals who had undergone AC and PZ.

**Fig. 2:**
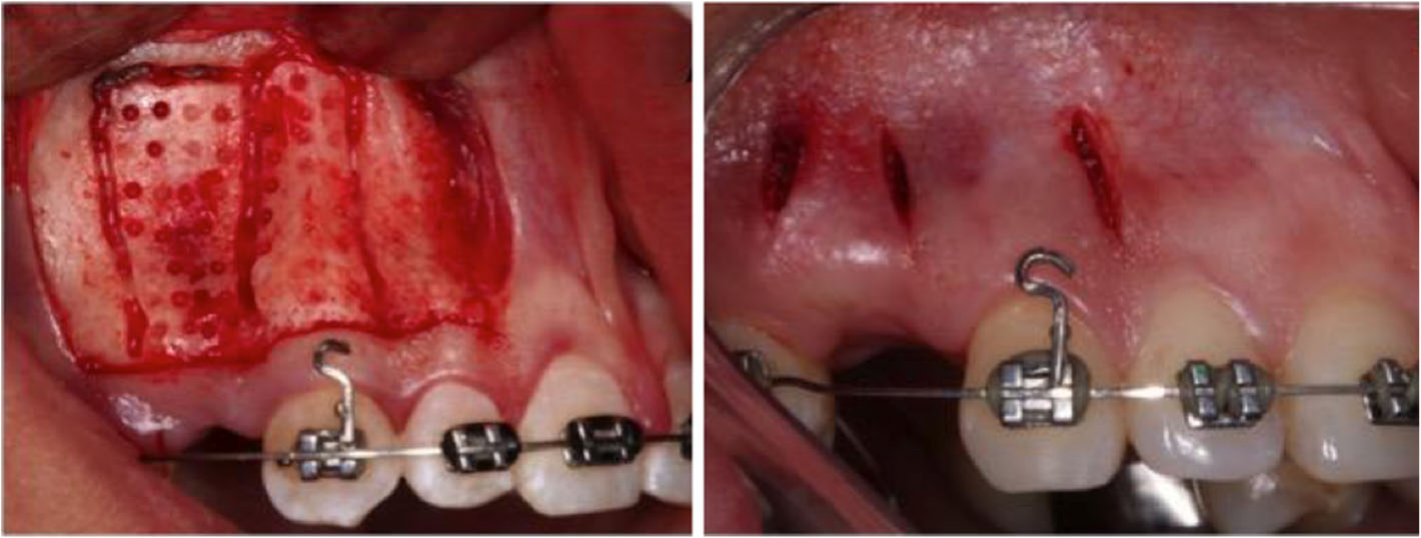
Surgical procedures. **A** Alveolar corticotomy; **B** piezocision

All surgical procedures were restricted to the buccal area and performed only once in the experimental side. To perform the AC, after local anesthesia, a full-thickness mucoperiosteal flap was performed from the mesial of the second premolar to the mesial of the lateral incisor (Fig. 2A). The incisions were performed without detachment of the papillae, with the intention of preserving them. Vertical corticotomies were performed in the mesial and distal regions of the canine root, as well as in the mesial of the second premolar. Above the apex of the canine, a horizontal corticotomy was performed joining the vertical cuts. Additional spherical bone injuries were done from the alveolar surface of the canine to the mesial of the second premolar. The perforations were limited to the depth of the cortical and performed with a spherical bur number 2 in low speed under copious irrigation with saline solution. The flap was repositioned and sutured.

For the piezocision procedure, after local anesthesia, vertical linear incisions were performed with a number 15 scalpel blade at the mesial and distal aspect of canine root, as well as mesially at the second premolar (Fig. 2B). The incisions began 5 mm above the papilla in order to preserve it, and extended parallel to the roots. After soft tissue incision, vertical bone injuries were performed with piezoelectric tips (SF3 insert Piezo DentSurg, CVDentus®, São José dos Campos, SP, Brazil) with a depth of 3 mm and a height of 5 mm. The incisions were not sutured, as suggested by the authors who described the technique [8].

Canine retraction started immediately after surgical or control interventions. A nickel-titanium spring (Sentalloy, Dentsply GAC, York, PA) connected the miniscrew to a post welded to the canine bracket, to allow application of the distalization force as close as possible to the canine’s center of resistance. Patients were evaluated at every 2 weeks and the retraction force (1.18 N) was verified with a dynamometer in each appointment (Correx, Haag-Streit AG, Koeniz, Switzerland).

Intraoral digital scans (TRIOS, 3Shape, Copenhagen, Denmark) were performed before (T0), 1 (T1), and 2 weeks (T2) after the beginning of canine retraction, and subsequently at every 2 weeks until a maximum period of 6 months (T13). Gingival crevicular fluid (GCF) samples were collected [14] with periopaper strips from the canine gingival crevice at T0, T1, T2, T3, T5, and T7, from the mesial and distal canine sites.

### Outcomes and any changes after trial commencement

**Fig. 3:**
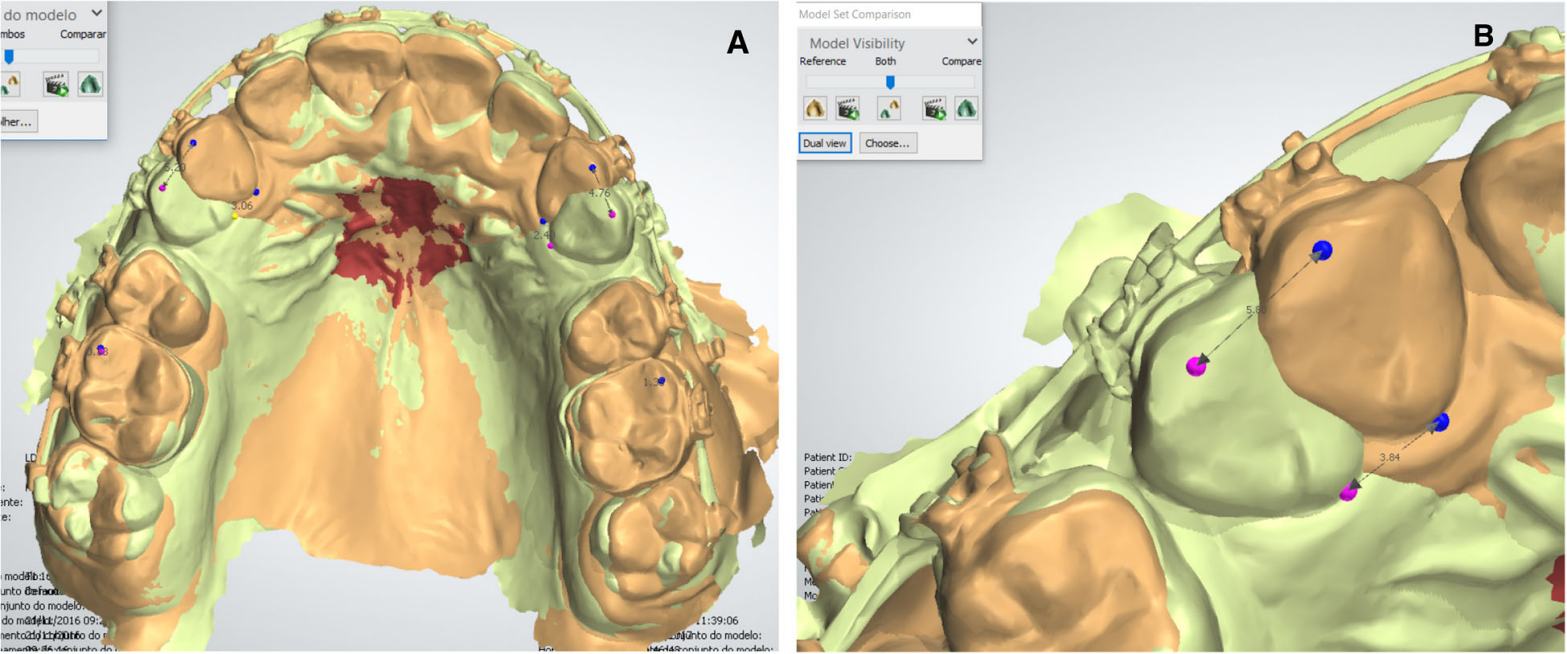
Superimposed digital models. In brown, the baseline model and in green a comparison model. **A** Occlusal view illustrating superimposition on palatal rugae. **B** Incisal and cervical measurement performed on the canines

The primary outcome was the cumulative moved distance of the maxillary canines. Measurements were performed using the digital model obtained on T0, using the OrthoAnalyser 2015 software (3Shape, Copenhagen, Denmark). The other digital models were superimposed with this reference model by matching the palatal rugae, since this structure is stable even during OTM [15–17]. The superimposition was performed using 3 points and one surface. The medial points of the 3rd rugae were bilaterally marked, as well as a 3rd point in another region of the third rugae. The surface was selected from the midpoint between the 3rd right and left rugae, with an expansion of 10 clicks. The surface selection is designated as 3D surface-to-surface matching (best-fit method). This superimposition method was considered accurate to allow 3D evaluation of OTM [18, 19]. To determine the retraction rate, the distances between the canines in the overlapping models were measured both cervically (center of cervical margin) and incisally (cusp tip) as shown in Fig. 3. This method was based in a previous study [9].

GCF biomarker levels were examined as a secondary outcome. Briefly, the paper strips were eluted with 150 μl of sterile of PBS and shaken at room temperature for 15 min. Strips were carefully removed from the tube, the eluate was centrifuged (5 min, 3000×*g*) and the levels of interleukin 1-beta (IL-1β), tumor necrosis factor-alpha (TNF-α), receptor activator of nuclear factor kappa-Β ligand (RANKL), osteoprotegerin (OPG), and Dickkopf-related protein 1 (DKK1) were determined on the Luminex-200 (Luminex, Austin, TX) using the human bone magnetic bead panel (EMD Millipore, Chicago, IL) following manufacturer’s instructions. Quantitative results (pg/ml) were generated with the Xponent software (Millipore Corporation).

### Sample size calculation, randomization, and blinding

The sample size was determined based on data from a previous study [20], which also had as primary objective the evaluation of OTM rate. Considering a power of 80% and a level of significance of 5%, a sample size of 15 individuals per group with a total sample of 45 individuals was required. The age range of the patients who participated in the study was necessary to reach the adequate sample size. Potential differences due to this age range are compensated by the split-mouth design. Prior to the start of the study, randomization by block (definition of the group and the decision of the experimental or control sides) was performed by Quick Calcs (GraphPad Software, Inc., La Jolla, CA) in a randomization center, by a person not associated to the patients’ recruitment center. Recruitment center individuals, including orthodontist and oral surgeon, did not have access to the allocation sequence, ensuring the concealment of allocation. The allocation (group and side) was informed to the oral surgeon by telephone immediately before each surgical procedure.

Blinding of the surgeon (R.G.H.) and participants was not possible. For the digital models’ measurements, the operator (D.F.F.) was not aware of the patients group, as well as whether the side was experimental or control. Digital models were previously coded, and they were also cut up to 1 or 2 mm above the teeth cervical margin in the buccal side in order to prevent visualization of possible surgical scars.

### Statistical analysis

The analyses were processed in the R software (version 3.4.2; R Foundation for Statistical Computing, Vienna, Austria). All variables referring to 50 randomized digital models were remeasured after 1 month and the intraclass correlation coefficient ranged from 0.94 to 0.99 showing excellent intrarater reliability. The paired t test did not detect a systematic error. Means and standard deviations were calculated for all variables. The generalized estimating equation (GEE) was used with a marginal log-linear regression to compare the amount of retraction between sides over time and to make comparisons in biomarker levels. The Gamma distribution was used to account for non-normality of the biomarker data. The level of significance was predetermined at 5%.

## Results

Fifty-one participants who met the inclusion criteria were randomly included. There were 4 patient dropouts: one, before allocation gave up any orthodontic treatment, and the other 3 moved to different cities and were unable to proceed with treatment. The participant’s flow during the trial is described in the CONSORT flow chart (Fig. 1). Therefore, the present study analyzed 47 participants that have a mean age of 20.7 years (15 to 38 years), of whom 28 were female. The follow-up occurred until March 2018. This data and the type of malocclusion in each group are described in Table 1.

**Table 1:** Patient’s baseline characteristics

		Group 1	Group 2	Group 3
Participants		16	16	15
Age (Mean and SD)		21.1 (7.14)	19.4 (6.70)	21.6 (6.56)
Gender	Male	5	8	6
	Female	11	8	9
Malocclusion	Class I	8	5	11
	Class II division 1	8	10	3
	Class II division 2	0	1	1
	Anterior crowding	7	6	5
	Deep bite	2	3	4
	Open bite	4	2	0
	Posterior crossbite	0	0	2
	Biprotrusion	4	1	7

**Fig. 4:**
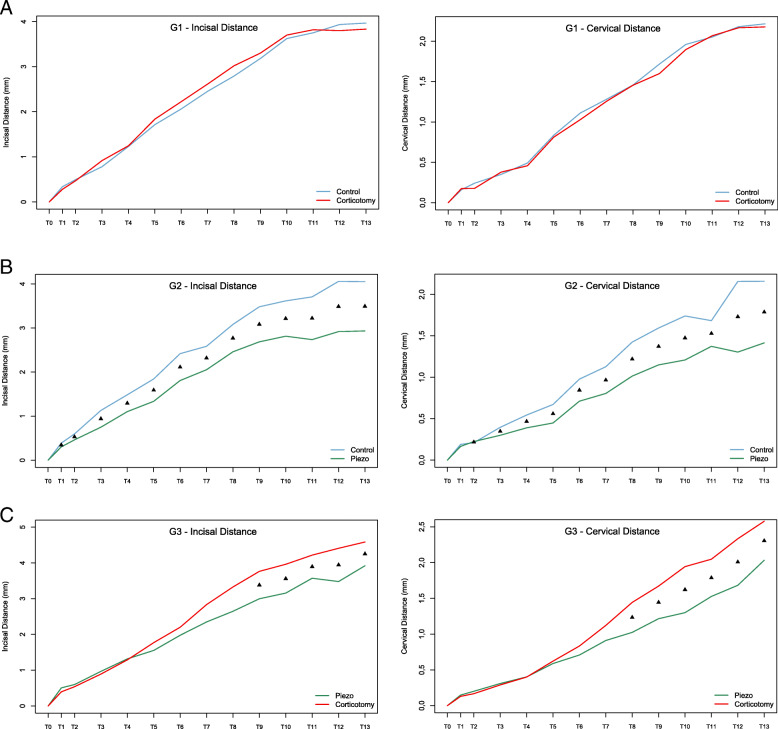
Cumulative mean changes of canine movement over time. **A**, G1; **B**, G2; **C**, G3 (▲, *P* < .05)

In G1, significant differences at any of the evaluated times for the incisal and cervical measurements between AC and control sides were not observed (*p* > 0.05) (Fig. 4A and Table 2). In G2, significant differences between PZ and control sides in incisal and cervical measurements were observed from T2 to T13 (*p* < 0.05), showing a constant tendency of a lower cumulative distance in the canines on the PZ side (Fig. 4B and Table 3). In G3, significant differences between AC and PZ from T9 for the incisal, and from the T8 for the cervical measurement sides were observed (*p* < 0.05), revealing a progressively lower cumulative distance on the PZ side (Fig. 4C and Table 4).

**Table 2:** Comparison of incisal and cervical accumulative moved distances (mm) between sides over time - group 1

Source		Incisal					Cervical				
		Mean	S.D.	Exp (β)	C. I.	*P* value	Mean	S.D.	Exp (β)	C. I.	*P* value
1 week	Control	0.34	0.14	1.00	-	-	0.16	0.12	1.00	-	-
	Corticotomy	0.28	0.13	1.03	[0.87; 1.23]	0.728	0.18	0.13	0.95	[0.78; 1.14]	0.574
2 weeks	Control	0.50	0.25	1.00	-	-	0.24	0.19	1.00	-	-
	Corticotomy	0.47	0.32	1.03	[0.87; 1.22]	0.716	0.18	0.12	0.95	[0.79; 1.14]	0.578
4 weeks	Control	0.78	0.41	1.00	-	-	0.35	0.18	1.00	-	-
	Corticotomy	0.92	0.28	1.03	[0.88; 1.22]	0.693	0.38	0.21	0.95	[0.80; 1.14]	0.590
6 weeks	Control	1.23	0.38	1.00	-	-	0.49	0.17	1.00	-	-
	Corticotomy	1.25	0.45	1.04	[0.88; 1.22]	0.672	0.46	0.25	0.96	[0.80; 1.14]	0.609
8 weeks	Control	1.71	0.35	1.00	-	-	0.84	0.25	1.00	-	-
	Corticotomy	1.84	0.83	1.04	[0.88; 1.22]	0.654	0.81	0.45	0.96	[0.81; 1.14]	0.636
10 weeks	Control	2.06	0.65	1.00	-	-	1.11	0.34	1.00	-	-
	Corticotomy	2.23	0.91	1.04	[0.88; 1.22]	0.640	1.03	0.48	0.96	[0.81; 1.15]	0.671
12 weeks	Control	2.45	0.86	1.00	-	-	1.28	0.39	1.00	-	-
	Corticotomy	2.62	1.02	1.04	[0.88; 1.23]	0.630	1.26	0.63	0.97	[0.81; 1.16]	0.710
14 weeks	Control	2.79	1.00	1.00	-	-	1.46	0.51	1.00	-	-
	Corticotomy	3.02	1.05	1.04	[0.88; 1.24]	0.625	1.46	0.62	0.97	[0.80; 1.17]	0.751
16 weeks	Control	3.18	0.90	1.00	-	-	1.72	0.55	1.00	-	-
	Corticotomy	3.30	1.22	1.04	[0.88; 1.24]	0.622	1.60	0.74	0.97	[0.80; 1.19]	0.792
18 weeks	Control	3.62	0.96	1.00	-	-	1.96	0.57	1.00	-	-
	Corticotomy	3.70	1.17	1.05	[0.87; 1.26]	0.623	1.90	0.65	0.98	[0.79; 1.21]	0.831
20 weeks	Control	3.75	1.11	1.00	-	-	2.05	0.73	1.00	-	-
	Corticotomy	3.82	1.17	1.05	[0.87; 1.27]	0.626	2.07	0.70	0.98	[0.78; 1.23]	0.867
22 weeks	Control	3.94	1.34	1.00	-	-	2.18	0.90	1.00	-	-
	Corticotomy	3.80	1.05	1.05	[0.86; 1.28]	0.631	2.17	0.87	0.98	[0.77; 1.25]	0.899
24 weeks	Control	3.97	1.41	1.00	-	-	2.22	0.95	1.00	-	-
	Corticotomy	3.83	1.00	1.05	[0.85; 1.30]	0.636	2.18	0.55	0.99	[0.76; 1.28]	0.928

*p* values were obtained by marginal log-linear regression. Level of significance = 5%; *, not significant (*p* > 0.05); *SD* standard deviation, *CI* confidence interval (95%), *Exp.* exponential

**Table 3:** Comparison of incisal and cervical accumulative moved distances (mm) between sides over time - group 2

Source		Incisal					Cervical				
		Mean	S.D.	Exp (β)	C. I.	*P* value	Mean	S.D.	Exp (β)	C. I.	*P* value
1 week	Control	0.39	0.21	1.00	-	-	0.19	0.15	1.00	-	-
	Piezo	0.30	0.17	0.74	[0.63; 0.86]	0.000*	0.17	0.12	0.83	[0.69; 1.00]	0.055
2 weeks	Control	0.60	0.34	1.00	-	-	0.21	0.12	1.00	-	-
	Piezo	0.46	0.28	0.74	[0.64; 0.85]	0.000*	0.22	0.13	0.82	[0.69; 0.98]	0.030*
4 weeks	Control	1.13	0.66	1.00	-	-	0.40	0.26	1.00	-	-
	Piezo	0.75	0.30	0.74	[0.64; 0.85]	0.000*	0.30	0.19	0.80	[0.68; 0.94]	0.006*
6 weeks	Control	1.48	0.77	1.00	-	-	0.54	0.31	1.00	-	-
	Piezo	1.10	0.54	0.74	[0.64; 0.85]	0.000*	0.39	0.25	0.78	[0.68; 0.90]	0.001*
8 weeks	Control	1.84	0.90	1.00	-	-	0.67	0.42	1.00	-	-
	Piezo	1.34	0.68	0.74	[0.65; 0.85]	0.000*	0.45	0.24	0.76	[0.67; 0.87]	0.000*
10 weeks	Control	2.42	1.09	1.00	-	-	0.98	0.50	1.00	-	-
	Piezo	1.81	0.89	0.74	[0.65; 0.85]	0.000*	0.71	0.31	0.74	[0.65; 0.84]	0.000*
12 weeks	Control	2.58	1.12	1.00	-	-	1.13	0.56	1.00	-	-
	Piezo	2.05	0.91	0.74	[0.65; 0.85]	0.000*	0.80	0.38	0.72	[0.63; 0.83]	0.000*
14 weeks	Control	3.08	1.26	1.00	-	-	1.43	0.61	1.00	-	-
	Piezo	2.46	1.13	0.75	[0.65; 0.85]	0.000*	1.01	0.51	0.70	[0.61; 0.81]	0.000*
16 weeks	Control	3.48	1.23	1.00	-	-	1.60	0.58	1.00	-	-
	Piezo	2.69	1.14	0.75	[0.65; 0.85]	0.000*	1.15	0.56	0.69	[0.59; 0.80]	0.000*
18 weeks	Control	3.61	1.21	1.00	-	-	1.74	0.83	1.00	-	-
	Piezo	2.81	1.33	0.75	[0.65; 0.86]	0.000*	1.21	0.58	0.67	[0.56; 0.80]	0.000*
20 weeks	Control	3.71	1.33	1.00	-	-	1.68	0.69	1.00	-	-
	Piezo	2.74	1.44	0.75	[0.65; 0.87]	0.000*	1.37	1.36	0.65	[0.54; 0.79]	0.000*
22 weeks	Control	4.06	0.97	1.00	-	-	2.16	0.75	1.00	-	-
	Piezo	2.92	1.16	0.75	[0.65; 0.87]	0.000*	1.30	0.63	0.64	[0.51; 0.79]	0.000*
24 weeks	Control	4.05	0.96	1.00	-	-	2.16	0.58	1.00	-	-
	Piezo	2.93	1.11	0.75	[0.64; 0.88]	0.000*	1.42	0.58	0.62	[0.49; 0.79]	0.000*

*p* values were obtained by marginal log-linear regression. Level of significance = 5%; *, significant (*p* < 0.05); *SD* standard deviation, *CI* confidence interval (95%), *Exp.* exponential

**Table 4:** Comparison of incisal and cervical accumulative moved distances (mm) between sides over time - group 3

Source		Incisal					Cervical				
		Mean	S.D.	Exp (β)	C. I.	*P* value	Mean	S.D.	Exp (β)	C. I.	*P* value
1 week	Corticotomy	0.40	0.16	1.00	-	-	0.13	0.09	1.00	-	-
	Piezo	0.51	0.30	1.11	[0.89; 1.37]	0.349	0.15	0.09	1.13	[0.84; 1.51]	0.422
2 weeks	Corticotomy	0.54	0.22	1.00	-	-	0.17	0.14	1.00	-	-
	Piezo	0.60	0.29	1.09	[0.88; 1.34]	0.441	0.20	0.13	1.10	[0.83; 1.46]	0.513
4 weeks	Corticotomy	0.89	0.39	1.00	-	-	0.29	0.15	1.00	-	-
	Piezo	0.97	0.46	1.04	[0.85; 1.27]	0.679	0.31	0.19	1.04	[0.80; 1.35]	0.754
6 weeks	Corticotomy	1.29	0.46	1.00	-	-	0.40	0.18	1.00	-	-
	Piezo	1.32	0.64	1.00	[0.83; 1.21]	0.982	0.40	0.24	0.99	[0.78; 1.26]	0.929
8 weeks	Corticotomy	1.77	0.68	1.00	-	-	0.62	0.24	1.00	-	-
	Piezo	1.55	0.64	0.96	[0.80; 1.16]	0.690	0.59	0.32	0.94	[0.75; 1.18]	0.585
10 weeks	Corticotomy	2.20	0.87	1.00	-	-	0.83	0.31	1.00	-	-
	Piezo	1.98	0.87	0.93	[0.77; 1.11]	0.399	0.71	0.39	0.89	[0.72; 1.11]	0.299
12 weeks	Corticotomy	2.83	0.92	1.00	-	-	1.12	0.54	1.00	-	-
	Piezo	2.35	0.89	0.89	[0.75; 1.06]	0.194	0.91	0.55	0.85	[0.68; 1.05]	0.126
14 weeks	Corticotomy	3.33	0.88	1.00	-	-	1.44	0.46	1.00	-	-
	Piezo	2.65	0.99	0.85	[0.72; 1.02]	0.080	1.02	0.47	0.80	[0.64; 1.00]	0.048*
16 weeks	Corticotomy	3.76	1.00	1.00	-	-	1.67	0.41	1.00	-	-
	Piezo	3.00	1.08	0.82	[0.69; 0.98]	0.029*	1.22	0.55	0.76	[0.61; 0.96]	0.019*
18 weeks	Corticotomy	3.96	0.68	1.00	-	-	1.94	0.55	1.00	-	-
	Piezo	3.16	1.19	0.79	[0.66; 0.94]	0.010*	1.30	0.69	0.72	[0.57; 0.92]	0.008*
20 weeks	Corticotomy	4.22	0.95	1.00	-	-	2.05	0.60	1.00	-	-
	Piezo	3.57	1.21	0.76	[0.63; 0.91]	0.003*	1.53	0.78	0.69	[0.53; 0.89]	0.004*
22 weeks	Corticotomy	4.41	0.92	1.00	-	-	2.33	0.71	1.00	-	-
	Piezo	3.48	1.08	0.73	[0.60; 0.88]	0.001*	1.68	1.00	0.65	[0.49; 0.86]	0.003*
24 weeks	Corticotomy	4.58	0.91	1.00	-	-	2.58	0.66	1.00	-	-
	Piezo	3.92	0.83	0.70	[0.57; 0.85]	0.000*	2.03	0.72	0.62	[0.45; 0.84]	0.002*

*p* values were obtained by marginal log-linear regression. Level of significance = 5%; *, significant (*p* < 0.05); *SD* standard deviation, *CI* confidence interval (95%), *Exp.* exponential

**Fig. 5:**
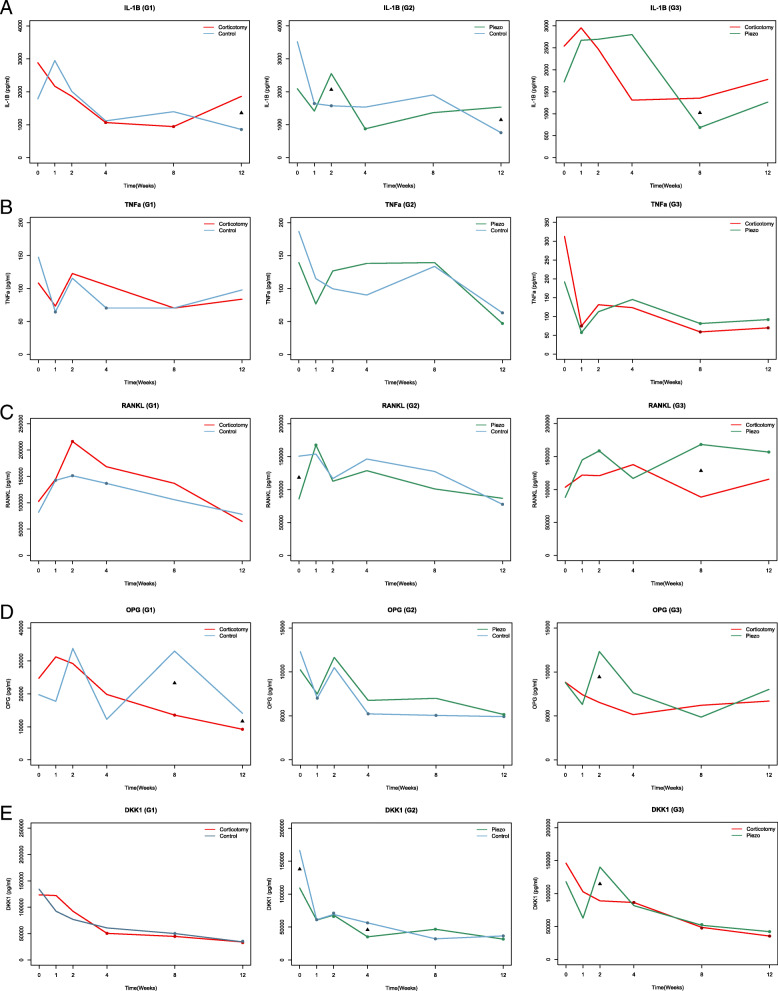
Biomarkers levels over time. **A**, IL1B; **B**, TNFa; **C,** RANKL; **D**, OPG; **E**, DKK1

In all groups, only isolated time-dependent significant differences in bone biomarker levels between sides were observed (Fig. 5). The analysis of biomarker levels in the GCF showed that IL-1β levels were higher on the intervention side in G1 (T7) and G2 (T2; T7) in comparison to the control side (*p* = 0.000; *p* = 0.000; *p* = 0.0012). When comparing the techniques (G3 group), IL-1β levels were higher (*p* = 0.033) on the AC side (T5) in comparison to the PZ side. TNF-α expression did not reveal significant differences between sides, exposed or not to the surgical procedures, in all groups. RANKL levels were higher only on the PZ side (T5) when compared to the AC side (*p* = 0.014; G3). OPG levels were lower on the AC side (T5 *p* = 0.006; T7 0.000) than in the control side in G1, whereas on the PZ side (T2; T7) were higher (*p* = 0.016) in comparison to the AC side in G3. DKK1 levels on the PZ side (T3) were lower (*p* = 0.033) than in the control side in G2, whereas on the PZ side (T2) were higher (*p* = 0.018) in comparison to the AC side in G3. The confidence interval was 95%.

## Discussion

The present data derived from a longer follow-up period (6 months), conducted 3D model superimposition and GCF bone biomarkers analysis, indicate that AC was not effective in accelerating canine retraction. This result differs from a previous that performed AC on the vestibular and palatal cortical bone [20]. Since RAP is proportional to the amount of bone injury [21], it is possible that this additional injury (i.e., palatal) might be necessary to accelerate OTM. Nevertheless, in their study measurements were obtained with a digital caliper directly in the mouth, and it is important to mention that the authors measured the rate of space closure, rather than canine retraction per se, and since the loss of anchorage may vary between the experimental and control sides due to the different bone metabolism rates, bias could be present [20].

Moreover, the present study used a power-arm soldered to the canine bracket in order to apply the force closer to the tooth’s center of resistance, and the previous carried out the application of force directly on the bracket, which could have caused greater inclination, higher rates of tooth movement, and less bodily movement [2, 20]. Another factor that may have contributed to a lower rate of canine retraction in our study comparing to the previous is the time when the extractions were performed. Starting retraction of the canine right after extractions could result in greater movement acceleration since extractions, by themselves, could be able to induce RAP and accelerate OTM [22]. We chose to perform the extraction before alignment and leveling to avoid two types of surgical trauma for the patient in the same day, and to evaluate the solely influence of AC and PZ in the rate of tooth movement during canine retraction.

Our results showed that PZ was not effective to accelerate the rate of canine movement. This result is in accordance to other trials evaluating different OTM [11, 12]. In contrast, a previous study described favorable results for PZ during canine retraction [9]. In the present study, the vertical extension of PZ was standardized at 5 mm, with a depth of 3 mm, as proposed previously [8], whereas the other extended the incision vertically by approximately 10 mm, and the retractions were performed by elastomeric chain [9], not by nickel-titanium coil springs, which made it difficult to standardize the applied forces. In fact, our results showed that PZ tended to slow the retraction. It is not possible to state the exact reason for piezocision to delay movement in the present study. Further animal and clinical trials may be needed to confirm these findings and investigate the biological plausibility.

Studies in humans have shown that cytokine levels in GCF change significantly over time during orthodontic movement [23, 24]. However, the comprehension of the effects of AC or PZ at the molecular level is still unclear. Since no distinct patterns were identified for any of the analyzed molecules, the biomarker data seems to reinforce the clinical data regarding the inability of AC and PZ to stimulate a significant distinct expression of these bone remodeling molecules during canine retraction. According to a previous study [25], when the force is applied to the tooth, the biological response of inflammatory mediators in GCF is not differentiated by type of movement, since this fluid presents free circulation in the gingival sulcus. Therefore, gingival crevicular fluid samples were collected from the mesial and distal canine sites in order to obtain a higher sample volume to allow the analysis of different biomarker levels. GCF samples were collected at the same period of the day in each patient. Nevertheless, it was not feasible to schedule the appointments and GCF collection in all patients at the same period of day, in order to minimize the possible interference of the circadian rhythm.

A limitation of this study is that these results may not be applicable to other types of OTM because we evaluated mesiodistal tooth movement through medullary bone. The lack of differences in canine retraction may be explained by the inability of cortical bone injuries to modulate RAP activation in the medullary bone, as suggested by an animal study conducted previously [26]. Thus, we hypothesize that AC and PZ may be better indicated in cases requiring movement toward the cortical bone, such as dento-alveolar expansions [8, 22]. Another limitation may be related to the occlusal contacts of the canines during the retraction. Nevertheless, articulating paper evaluation was performed at each visit and if necessary, correction was implanted. A .016″ × .022″ wire was used, which allows a certain degree of inclination because it has a smaller vertical section. Thicker wires would decrease this tendency; however, they would increase the coefficient of friction. Thus, in an attempt to minimize tipping during retraction, power arms were used.

No serious harms were observed during the research and treatments. Only one patient presented bone sequestration associated with piezocision, resolved without major intercurrences. The evaluation of root resorption was not the aim of this study. In principal, the decrease in bone density would reduce a possible accumulation of excessive pressure in the periodontal ligament and subsequent occurrence of root resorption. However, there is no consensus between the studies and more investigation is necessary [27–29]. Based on the ineffectiveness shown in our study and costs of the AC and PZ, the recommendation of these surgeries to accelerate the canine retraction is questionable.

## Conclusions

Based on this randomized clinical trial, corticotomy and piezocision appeared to be not effective to accelerate canine retraction and did not induce distinct patterns on biomarker expression in GCF. However, these findings cannot be generalized to other types of orthodontic movements, since only the canine distalization movement was investigated in the present study.

## Data Availability

All data generated or analyzed during this study are included in this published article.
